# The Role of Microparticles in Rheumatic Diseases and their Potentials as Therapeutic Tools

**Published:** 2016-07-19

**Authors:** Wen-Hai Shao

**Affiliations:** Division of Immunology, Allergy and Rheumatology, University of Cincinnati, Medical Science Building, Room 7410, 231 Albert Sabin Way, Cincinnati OH 45267, USA

**Keywords:** Microparticles, SLE, RA, Biomarker

## Abstract

Microparticles (MPs) play important roles in intercellular communication, including adhesion, signal transduction, cell activation, and apoptosis. They possess a wide spectrum of biological effects in the immune responses. MPs could be immunotolerogenic or immunogenic depending on the contents and composition. Elevated levels of MPs have been reported in many forms for rheumatic diseases. This review focuses on the immunopathogenic and therapeutic role of MPs in rheumatic diseases.

## Introduction

Microparticles (MPs, also known as microvesicules or ectosomes) are heterogeneous subcellular vesicular particles (0.1–1.0 nm in diameter) released constitutively from cells and platelets undergoing cell activation or cell death by blebbing or shedding [1,2]. Platelet MPs are usually the most abundant type in blood. The presence of basal levels of MPs is common in healthy individuals, and is estimated, in peripheral blood, to range between 5 and 50 g/ml (10^5^–10^6^ MPs/ml) [3]. Numerous types of MPs have been characterized with important physiologic effects by the detection of different cell surface antigens reflecting their origin and activation method.

MPs represent distinct subcellular structures and serve a prominent role in homeostasis and intercellular communication including immune activation. They can transfer bioactive molecules from parental to target cells, allowing for regulation and amplification of several biological mechanisms such as activation, apoptosis, coagulation, and proliferation. They can be released actively at early stages of apoptosis and emerge preferentially from regions of the membrane containing lipid rafts where accessory proteins are sorted with specific function [2]. Studies on patients with a wide range of rheumatic disease show increased MP numbers in blood [4]. MPs are known to display diverse pro-inflammatory and pro-thrombotic activities that can influence the course of rheumatic diseases such as systemic lupus erythematosus (SLE) and rheumatoid arthritis (RA). They can thus impact on the pathogenesis of rheumatic disease and serve as biomarkers of underlying cellular disturbance.

## Microparticles in Immune Tolerance

MPs may reposition nuclear constituent in a form that may be more accessible to the immune system. Free RNA is sensitive to RNase digestion and free DNA is abundant in the blood but inactive. The incorporation of DNA and RNA into the lipid complexes of MPs increases the stability of the nucleic acids by protecting them from degradation. The bone marrow is a site of extensive cell turnover. The central tolerance to self-nucleic components might partially owe to the enhanced capacity of MPs to present DNA/RNA in the bone marrow during B cell development. Their special structure and membrane components may make MPs more effective in inducing central B-cell tolerance through clonal deletion by presenting autoantigens to immature B cells, along with signalling through non-TLR sensors (reviewed in [5]). The special membrane components and intercellular function of MPs could also facilitate the presentation of autoantigens (i.e. DNA/RNAs) to B cells through macrophages and DCs to induce B-cell anergy. In the periphery, MPs in healthy individuals may serve to continuously anergic induction the immune system with autoangtigens to avoid self-activation.

A number of MPs generated from apoptotic and activated cells/platelets are phosphatidylserine (PS) positive [6]. PS is the commonly used marker to detect apoptotic cell-derived MPs. PS can also be detected through membrane receptors or bridging molecules, including milk fat globule EGF factor 8 (MFG-E8), growth arrest specific protein 6 (GAS 6), and Protein S. They are in turn recognized by their cell surface receptors on phagocytes, such as αVß5 and TAM (Tyro-3, Axl, and Mer) receptor tyrosine kinases [7–9]. This process not only facilities apoptotic cell clearance, but also helps to maintain immune homeostasis. MPs may utilize this mechanism to maintain the peripheral immune tolerance. Taken together, MPs may actively participate in the maintenance of immune homeostasis and tolerance.

## Pathogenesis of Microparticles in Rheumatic Disease

### Pathogenesis

Under certain circumstances (i.e. genetic predisposition, defects in apoptotic clearance and viral/bacterial infection), MPs may present auto antigens to APCs in an immunogenic way. Studies on patients with a wide variety of rheumatic diseases have demonstrated significant elevations in circulating MPs compared to control populations. MPs display diverse pro-inflammatory and pro-thrombotic activities that can influence the course of rheumatic diseases and may serve as potentially important mediators of disease pathogenesis. SLE patients have elevated type I interferon (IFN-I) and IFN-inducible gene expression (IFN signature) [10]. Activation of the IFN-I pathway is believed to be crucial for the proinflammatory state in SLE. Recent study from Niessen et al. revealed a synergistic effect of MPs and IFN-. The combination of apoptotic-cell-derived MPs and IFN-α resulted in enhanced monocyte phagocytosis with increased pro-inflammatory cytokine (TNF, IL-6, IL-8) secretion [10]. Particles from patients with RA and SLE may bear substantial amounts of IgG (Immunoglobulin G) and complement components on their surfaces. Furthermore, studies using both mAbs as well as patient plasma indicate that anti-nucleosomal antibodies can bind to particles generated *in vitro* by apoptotic cells [11,12]. In SLE, the amount of this antigenic material may increase because of excessive cell death and impaired clearance of apoptotic cells. Apoptotic cells not being cleared in a timely manner often release dangerous signals that lead to proinflammatory response [13]. Under such conditions, nucleic acids incorporated in microparticles may have particular potency in stimulating inflammatory responses. In this regard, MPs may directly impact cellular immune responses underlying SLE, shifting tolerogenic immune responses to immunogenic responses.

MPs may also contribute to the pathogenesis of rheumatic diseases by the formation of immune complexes. As components of immune complexes, DNA and RNA are rendered more stable and are protected from DNases and RNases, respectively. DNA and RNA could potentially stimulate immune responses, especially when in the form of immune complexes. Lupus plasma contains MPs with IgG binding properties and the number of IgG-positive particles was correlated with anti-DNA levels [12]. Nielsen et al. studied MPs from 68 well-characterized SLE patients and found significantly increased total and relative numbers of IgG-positive MPs with a significantly increased load of IgG, IgM, and C1q per MP in SLE patients compared to healthy controls. IgG-positive MPs were significantly associated with the presence of anti-dsDNA autoantibodies [14]. These studies support the notion that MPs carry significant amounts of autoimmunogenic material, which may enhance any existing immunostimulation. Ig-containing MPs may also contribute to the systemic complement activation observed in SLE and provide adhesion (through Ab-Ag interaction) and costimulatory molecules (through complement activation) that result in IC deposition when binding to various cells. MPs found in SLE plasma may, on the other hand, compete with apoptotic cells for the PS receptor on macrophages, reducing phagocytosis of apoptotic cells, consequently resulting in secondary necrosis and aggravating the existing pathological conditions. Supporting this notion is the study showed that MPs prepared from apoptotic Jurkat cells inhibited the phagocytosis of apoptotic cells by THP-1 macrophages in a dose-dependent manner [3].

During activation and apoptosis, the contents of cells undergo extensive modification, degradation, and translocation. As shown in other studies, particles from various sources may differ in functional properties. MPs in rheumatic diseases may contain similar amounts of DNA/RNA compared to normal controls, but the modification of nucleic acids may make them potential immunogens and provoke cytokine production [14]. Several epigenetic alterations have been suggested to favor the development of anti-nucleosome autoantibodies [15]. Although native DNA is a weak immunogen, reactive oxygen species (ROS)-modified DNA is immunogenic and is recognized by anti-dsDNA antibodies isolated from lupus patients [16]. DNA methylation is another epigenetic modification that might be associated with an increased immunogenicity [17,18]. Finally, apoptotic DNA cleavage might itself be considered an epigenetic modification, though the immunogenicity of such modification has not been studied.

Increased amount of MPs can also occur in synovial fluid, where MP levels can far exceed those of blood level. MPs can act locally to drive synovitis and systemically to promote vascular disturbances. In the study of 19 patients, platelet-derived MPs from plasma of clinically active as well as inactive patients with RA were higher than those of healthy controls and levels of PMPs (platelet-derived MPs) also correlated with disease activity [19]. Vinuela-Berni et al. revealed a significant positive correlation between the levels of MPs and DAS28 (Disease Activity Score in 28 joints) [20]. Joint fluid MPs may drive cytokine production and activate synoviocytes locally in SLE and RA. MPs from fluids of RA patients incubated with fibroblast-like synoviocytes (FLS) induced release of inflammatory cytokines (IL-6 and IL-8) and chemokines (MCP-1 and RANTES). MPs isolated from RA patients with high DAS28 levels enhanced release of IL-1, IL-17, and TNF-a [20]. Moreover, when synovial FLS were incubated with autologous MPs, increased levels of MCP-1 and IL-8 were observed [21]. MPs can also activate RA synovial fibroblasts to selectively release pro-angiogenic ELR+ (glutamic acid-leucine-arginine) chemokines, without affecting proliferation and viability [22]. Particles in synovial fluid can drive synovial fibroblasts to produce matrix metalloproteinases, a critical feature of joint destruction in RA, as well as chemokines by a mechanism dependent on NF-B [23]. When cultured with MPs from T cells, synovial fibroblasts from RA patients up-regulated cyclooxygenase 2 (COX-2) and prostaglandin E2 (PGE2) expression and activated NF-B, AP-1, p38, and JNK pathways [24]. MPs in the joint may derive from monocytes and granulocytes. In a study with 10 RA patients from Netherlands, Berckmans et al. reported that LMPs (leukocytes-derived MPs) were strongly procoagulant via the factor VII-dependent pathway, which may contribute to the local hypercoagulation and fibrin deposition in inflamed joints of RA patients [25]. It’s worth to point out that recent literature suggests a mixture of evidences regarding number of microparticles from plasma and synovial fluid. A systemic review of clinical conditions must be considered when comparing MPs in plasma versus synovial fluid.

Although the correlation of increased MP numbers with rheumatic disease suggests a role in immunopathogenesis, elucidating the mechanism is difficult. First, MPs are heterogeneous populations, and different studies focus on diverse subpopulations of MPs, e.g. PS+ *vs*. PS-, endothelial derived, monocyte derived, lymphocyte-derived, platelet-derived et al. Second, studies have shown a paradoxical relationship of MPs to disease activity, which may be at least partly influenced by methodological differences. Third, MPs may bind to target cells or be sequestered within tissue compartments, especially under chronic inflammatory conditions found in autoimmune diseases. Fourth, increased phospholipase activity in autoimmune diseases may lead to increased lysis of MPs. Lastly, discrepancies between the MP studies in SLE and other rheumatic diseases could be partly explained by differences in patient selection and small numbers of patients in some studies. It may also reflect the lack of standardization of MP analyses and our current insufficient understanding of the complexity of the biology of MPs, including their tissue turnover and their exact role in homeostasis. The mechanisms of shedding MPs remain poorly understood, although cytoskeleton rearrangement and calcium signalling are involved in the process. Discriminating between MPs that arise through apoptosis versus activation and characterization of the MP antigen composition is an important goal of future studies.

## Mps in other Autoimmune Diseases

Most research of MPs in autoimmune diseases focused on SLE and RA. Few studies investigated MP levels in other forms of autoimmune disease. Sellam et al. investigated the plasma levels of total, platelet, and leukocyte derived MPs by prothrombinase capture assay and flow cytometry in 43 primary Sjögren's syndrome (pSS) patients and 44 healthy controls [26]. Patients with pSS showed significantly increased plasma levels of total MPs (ρ<0.0001). When compared to the MP levels in SLE and RA patients, levels of leukocyte-derived MPs were only increased in pSS individuals. Nevertheless, platelet-derived MP levels were inversely correlated with levels of serum β2 microglobulin, a marker associated with extra-glandular involvement [26]. A recent study investigated circulating endothelial microparticles (EMPs) (CD31+/CD42−) levels in 34 pSS patients and 18 age- and sex-matched controls. Similar significant differences of increased EMPs were observed in pSS patients with respect to healthy controls [27]. Takeshita et al. measured the concentration of MPs in blood samples from 53 psoriasis patients and 41 controls [28]. Significantly higher concentrations of endothelial-, platelet-and monocyte/macrophage-derived microparticles were found in psoriasis patients compared with controls. Relapsing-remitting patients showed the highest levels in the three subtypes (platelets, total leukocytes and monocytes) of MP [29]. A study involving 95 multiple sclerosis (MS) patients including all clinical forms (clinically isolated syndrome, relapsing-remitting, and secondary progressive and primary progressive) reported increased plasma levels of platelet-derived and endothelium-derived MPs [30]. MPs from relapsing-remitting MS patients induced a stronger disruption of endothelial barriers (measured by electric cell-substrate impedance sensing) than those from healthy donors or from patients with clinically isolated syndrome. Findings indicated that MPs in MS patients not only function as a biomarker but also play an active pathological role in increasing endothelial permeability and leukocyte infiltration, thus contributing to MS progression. Accordingly, plasma levels of EMP were significantly reduced following initiation of treatment with IFN-β1a in MS patients [31].

## Microparticles as Biomarkers in Rheumatic Diseases

MPs have received increased attention as universal markers of activation in eukaryotic cells. They carry markers of their parent cells, including those induced by activation, apoptosis, cell lysis, or oxidative stress. These properties permit detection of specific subpopulations. Increased neutrophil-, endothelial-, and platelet-derived MPs have been described in rheumatic diseases. MPs can provide important information about ongoing pathogenic processes that might be valuable clinically for diagnosing, accessing disease activity, and evaluating the effects of treatment.

EMPs are produced by endothelial cells in response to a variety of triggers and may act as biomarkers for endothelial activation and damage. Active SLE is associated with increased endothelial damage. Damaged endothelial cells often release EMPs into the blood. EMPs were significantly elevated in ANCA-(anti-neutrophil cytoplasmic antibody) associated small vessel vasculitis (AAV) [32]. Reduction in disease activity in SLE patients treated with immunosuppressive therapies was found to be associated with significant reduction in CD31+/annexin V+ CD42b- EMPs [33]. Hsu et al. demonstrated that inhibition of BTK (Bruton’s Tyrosine Kinase) activation attenuated collagen-induced production of PMPs. Decreased PMPs could reduce cytokine production and vascular abnormalities [34].

MPs may serve as biomarkers to define subsets of RA patients and provide information for disease activity and prognosis. Ostergaard et al. identified 531 unique circulating proteins from SLE patients and showed highly statistically significant differences of 248 proteins compared to healthy controls. SLE MPs showed an unique protein profile that could be distinguished from RA and SSc and from diseases controls [35]. MPs from those SLE patients were found to have laden with Ig and complement-specific proteins tagged for removal [35]. Encouraging results from Rodriguez-Carrio et al. showed a significant association between MP subsets and disease features in RA patients (EMP counts: disease duration GMP (ganulocyte-derived MP): DAS28 (disease activity score 28 points); and MoMP (monocyte-derived MP): RF (rheumatoid factor)[36].

However, a study from Van Eijk et al. reported that circulating MPs associated with complement activation were not affected despite intensive anti-inflammatory therapy in early RA patients [37]. Crookston et al. also found no difference in total MPs in SLE patients (n=51) relative to matched controls (n=21), but noted a significant reverse correlation between concentrations of monocyte-derived MPs and neuropsychiatric SLE activity [38]. Consistent with this study, no significant difference in levels of EMPs in SLE compared to healthy donors was reported by other groups [39,40]. Further experiments are needed to solve this discrepancy.

## The Therapeutic Prospect of MPs in Rheumatic Diseases

Current treatment options for autoimmune diseases often involve nonspecific immunosuppression that may result in enhanced patient susceptibility to opportunistic infections. The number of MP therapies is growing, recent advances in understanding the function of MPs in homeostasis and inflammation have reinforced the potential of MP therapies in controlling inflammation while restoring the immune tolerance in autoimmune disease. Micro-mediated drug delivery system, including polymeric particles, liposomes, and hydrogels are well established as methods for sustained release of therapeutics [41]. A number of microparticle-based approaches have been designed to induce tolerance or deliver therapeutic drug in more sustainable ways [42]. Artificial MP-based therapy is current under development and data are encouraging. Comparing to the biological MPs elaborated in this review, artificial MPs are easy to manipulate and homogeneous population is often assured.

Getts et al. coupled myelin proteolipid protein (PLP) epitope to carboxylated 500 nm polystyrene beads (PSB) and successfully induced tolerance with the prevention of experimental autoimmune encephalomyelitis (EAE) [43,44]. Importantly, treatment with the same PSB-PLP MPs at the first sign of disease also prevented initiation in the vast majority of mice. When the authors attached the PLP onto the US Food and Drug Administration-approved biocompatible, biodegradable negatively charged poly (lactide-co-glycolide) (PLG) microparticles, similar results were achieved. Intravenous administration of PLP-PLG was able to treat ongoing EAE and reduce the severity of relapse symptom [43]. The tolerogenic effects persisted for the duration of the mouse studies and depended on MARCO-bearing macrophages, which uptake the MPs and in turn lead to a long-term T cell-mediated tolerogenic response specific to the peptide antigen [45]. MPs induced T-cell tolerance may have broad therapeutic utility but the approach is challenged by the necessity to identify a defined T-cell epitope. This requirement may limit its role in the induction of tolerance in etiology-unknown autoimmune disease like SLE. However, glucocorticoids (GCs) encapsulated liposomes could ameliorate EAE to the same extent as free GC, but at strongly reduced dosage and application frequency [46].

Micro particle systems that selectively deliver drugs to inflamed synovium have the potential to improve drug efficacy while leaving extra-synovial tissues unaffected. Liposomal use has been widely studied as a potential carrier system for drug delivery for RA. An early study using liposome-encapsulated clondronate, an anti-inflammatory therapy that reduces bone resorption, resulted in a halt in disease progression and a reduction in inflammation [47]. Similarly, liposomal methotrexate conjugated to the y-carboxylic acid residue yielded a significant reduction of established joint inflammation [48].

## Conclusion and Future Direction

MPs consist of a communication network in transducing intercellular signals and maintaining the system bio physiological homeostasis. They display important biological properties that can mediate disease pathogenesis and provide important information of on-going pathogenic process. Though a consensus protocol to measure MP does not yet exist, most studies showed markedly altered concentrations and distributions of MPs subpopulations in patients with rheumatic diseases. In addition, the impact of circulating MPs is not well understood. However, MPs mediated drug delivery system could prolong drug retention time, increase patient compliance as well as therapeutic effect. Sustained therapeutic drug concentrations can also be achieved. The use of MPs as immune tolerogenic and as therapeutic agent has only recently initiated and is rapidly expanding. The design and safety of MPs will have to be addressed to achieve desirable outcome. The use of antigen-conjugated MPs appears to be therapeutically promising in experimental animal models [49]. An improved understanding of multifunctional complex network of MPs may prove critical for designing more effective therapeutics for rheumatic diseases.
